# Endovascular Crossing of Chronic Total Occlusions Using an Impulse: An Explorative Design Study

**DOI:** 10.1007/s13239-017-0306-1

**Published:** 2017-05-17

**Authors:** Aimée Sakes, Marleen van der Wiel, Dimitra Dodou, Paul Breedveld

**Affiliations:** Department of Biomechanical Engineering, Faculty of Mechanical, Maritime and Materials Engineering, Delft University of Technology, Mekelweg 2, 2628 CD Delft, The Netherlands

**Keywords:** Chronic Total Occlusions (CTO), Percutaneous Coronary Interventions (PCI), Medical device design, Cap puncture, Crossing

## Abstract

In this study we investigated whether exerting an impulse on a Chronic Total Occlusion (CTO) improves the success rate of CTO crossing as compared to the currently used method of statically pushing the guidewire against the CTO. A prototype (Ø2 mm) was developed that generates translational momentum using a spring-loaded indenter and converts it to an impulse during impact. Mechanical performance was evaluated by measuring the peak force and momentum for different spring compressions and strike distances in air and blood-mimicking fluid. Puncture performance, in terms of number of punctures, number of strikes to puncture, and energy transfer from the indenter to the CTO, was assessed for six tip shapes (stamp, wedge, spherical, pointed, hollow spherical, and ringed) on three CTO models with different weight percentages of gelatin and calcium. As a control, a Ø0.4 mm rigid rod was tested. A maximum indenter momentum of 1.3 mNs (velocity of 3.4 m/s), a peak force of 19.2 N (vs. 1.5 N reported in literature and 2.7 N for the control), and CTO displacement of 1.4 mm (vs. 2.7 mm for the control) were measured. The spherical and ringed tips were most effective, with on average 2.3 strikes to puncture the most calcified CTO model. The prototype generated sufficient peak forces to puncture highly calcified CTO models, which are considered most difficult to cross during PCI. Furthermore, CTO displacement was minimized, resulting in a more effective procedure. In future, a smaller, faster, and flexible clinical prototype will be developed.

## Introduction

During Percutaneous Coronary Interventions (PCI), a small guidewire (Ø0.36 mm) is gradually driven through the vasculature from an incision point in the groin or wrist towards an occlusion in the coronaries. Once arrived at the occlusion, a static (axial) load is applied on the guidewire by the interventional cardiologist from outside the body to puncture and cross the occlusion, after which the occlusion is reopened using a balloon catheter. Coronary Chronic Total Occlusions (CTOs), defined as heavily calcified, complete coronary occlusions of over 3 months old, represent the most challenging lesion type to be crossed during PCI, requiring a high skill level of the interventional cardiologists.^1^ The development and use of several dedicated guidewires, such as the *Confianza Pro* (Asahi Intecc, Nagoya, Japan) and the *Progress 200T* (Abbott Vascular, Abbott Park, IL), dedicated crossing and support catheters, such as the *Tornus* (Asahi Intecc, Nagoya, Japan) and the *Crossboss* (Boston Scientific, Natick, MA), crossing tools, such as the *Frontrunner XP* (Ø0.76–1 mm; Cordis Corporation, Miami, FL), the *Crosser Catheter* (Ø0.6–1.5 mm; BARD Peripheral Vascular Inc., Tempe, AZ), and the *Truepath* (Ø0.43 mm; Boston Scientific, Natich, MA), and crossing strategies (see Sakes *et al*.^2^ for a comprehensive overview) have contributed to a steady increase in the technical success rate of PCI in CTOs (i.e., the ability to cross the CTO and to successfully reopen the artery), as well as in the overall procedural success rate of these interventions (i.e., the proportion of procedures with no nosocomial major adverse cardiac events).^3^ However, the overall procedural success rate is still undesirably low. Whereas experienced operators can achieve success rates of up to 90%, success rates of experienced operators not specialized in CTO PCI are lower than 55%.^4^ The success rate is further depended on the characteristics of the CTO, of which age, increasing occlusion length, tortuosity, and cap ambiguity are historical predictors for technical and procedural failure, and to a lesser extent the used crossing technique (i.e., antegrade, retrograde, or a combined technique).^3^,^5^

Failing to cross heavily calcified CTOs intraluminally (i.e., crossing through the original coronary lumen and CTO body) with a guidewire accounts for approximately two-third of PCI failures and is mainly the result of guidewire buckling.^6^ Guidewire buckling occurs because the required puncture force of the CTO often exceeds the maximum load that the guidewire tip can sustain. Furthermore, the static load applied on the CTO may lead to displacement of the CTO up to 4 mm (estimated based on Thind *et al.* 7). Such a large displacement not only leads to energy dissipation, thereby reducing the maximum force that can be delivered on the CTO, but also increases the chance of blood vessel wall damage due to stretching.

Guidewire buckling can be prevented by increasing the critical load of the guidewire, by decreasing the penetration load of the CTO or by bypassing the CTO using so-called dissection re-entry techniques. In the latter case, the CTO is crossed *via* the much softer blood vessel wall. Because this method damages the blood vessel wall and re-entry is often challenging,^8^ we will focus on intraluminal crossing methods instead. Most clinically applied methods of preventing guidewire buckling aim to increase the critical load of the guidewire. This can be achieved by encompassing the guidewire with a support catheter or by employing a second device as support, such as a balloon catheter (balloon anchoring technique) or a guidewire (wire anchoring technique).^5^ Despite these measures, crossing difficulties (mainly due to guidewire buckling) are still observed.^5^

**Figure 1:**
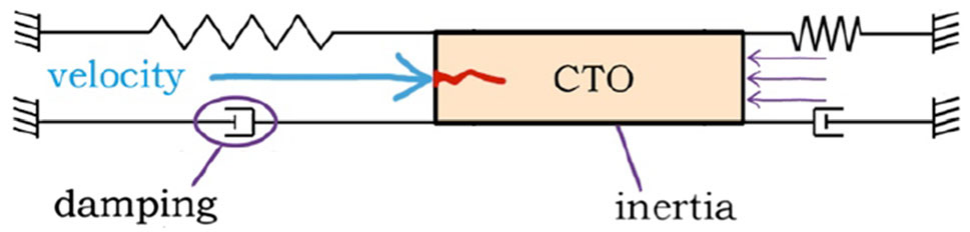
Impulse crossing method. The guidewire or crossing tool (blue arrow) collides with the CTO (orange), converting translational momentum to an impulse during impact, which in turn punctures the CTO (red jagged line). Due to the high velocity of the crossing tool, the inertia of the CTO and the damping of the blood vessel wall deliver the majority of the reaction force (purple arrows) and thus minimize CTO displacement and energy dissipation to the blood vessel wall (indicated by the spring and damper combination).

To increase the buckling resistance of the guidewire and prevent the energy dissipation associated with CTO displacement, a possible solution could be to use a dynamic loading method, in which a moving guidewire or dedicated CTO device would collide with a stationary CTO, converting translational momentum of the guidewire to an impulse exerted on the CTO. Here, momentum is defined as mass (*m* [kg]) × velocity (*v* [m/s]) of the guidewire ($p [{\text{Ns}}] = mv$), and impulse is defined as the integral of the impact peak force (*F*_*peak*_ [N]) over the time interval *dt* [s] during which it acts ($J [{\text{Ns}}] = \mathop \smallint \nolimits F_{peak} dt)$. Due to the (high) velocity of the guidewire, the damping of the blood vessel wall and the inertia of the CTO act as a “counterforce” to the impulse, and as such restrict the displacement of the CTO and the associated energy dissipation (Fig. 1). Furthermore, the critical buckling load of the crossing guidewire under dynamic impulse loading condition increases with decreasing load duration.^9^

The goal of this study was to explore the use of dynamic impulse loading for puncturing heavily calcified coronary CTOs. For this purpose, a prototype puncture tool able to deliver an impulse onto a CTO was designed and experimentally tested.

## Design

### Tip Design

To define the prototype diameter required for atraumatic navigation through the vascular system, the diameters of the arteries that potentially need to be crossed during PCI from the incision point to the lesion site were analyzed based on angiographic and ultrasound data in Refs. 10–16. Coronary CTOs are found in the Right Coronary Artery (RCA), Left Anterior Descending (LAD), Left Circumflex (LCx), and Left Main Trunk (LMT).^5^ In order to reach the coronary CTO, two main approaches can be taken^17^: (1) the radial approach, in which the CTO is reached *via* the radial artery in the wrist, and (2) the femoral approach, in which the CTO is reached *via* the common femoral artery. The smallest artery that needs to be crossed to reach the CTO is the RCA, which has a minimum diameter of 2.2 mm.^12^ Accordingly, the maximum prototype diameter was set to 2 mm.

The prototype tip consists of three components: (1) an actuation component that transfers the translational momentum through the instrument shaft towards the tip, (2) an indenter that applies an impulse onto the CTO, and (3) a reload mechanism that reloads and releases the actuation component.

### Actuation Component

**Figure 2:**
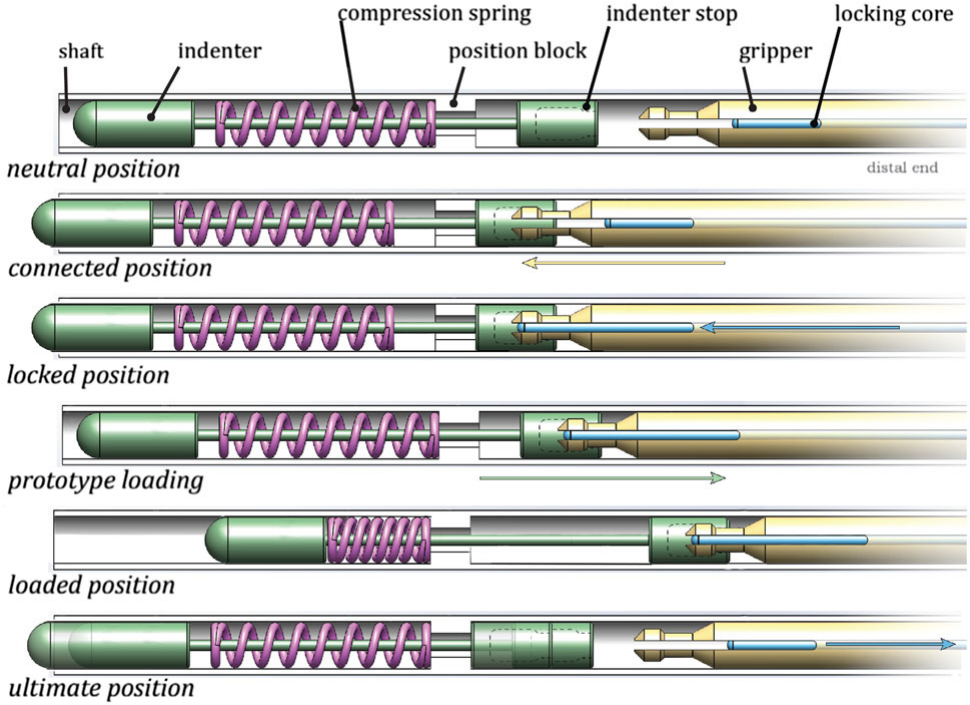
Tip design. The tip consists of five main parts: an outer shaft (grey), an indenter (green) with indenter stop, a compression spring (purple), a position block (white), and a reload mechanism (gripper: yellow and locking core: blue). Row indications: (1) Neutral position, showing the spring (purple) in rest (free length). (2) Connected position, showing the gripper inserted into the indenter stop. (3) Locked position, showing the gripper locked into place into the indenter stop by moving the locking core forward. (4) Spring compression by pulling the load mechanism backwards. (5) Loaded position, showing the spring being compressed by translating the load mechanism with the indenter stop backwards. (6) Ultimate position, showing the indenter being released and accelerated forward by the compression spring until the indenter stop reaches the position block.

The actuation component consists of a compression spring located at the distal tip of the instrument. Translational momentum is generated by compressing the spring with a load mechanism and a position block (Fig. 2). By varying the spring compression distance, the momentum (*p*) of the indenter can be adjusted.

### Indenter

The indenter receives the momentum generated by the compression spring and subsequently applies an impulse onto the CTO. The indenter consists of two parts: the indenter tip and the indenter stop.

#### Indenter Tip

**Figure 3:**
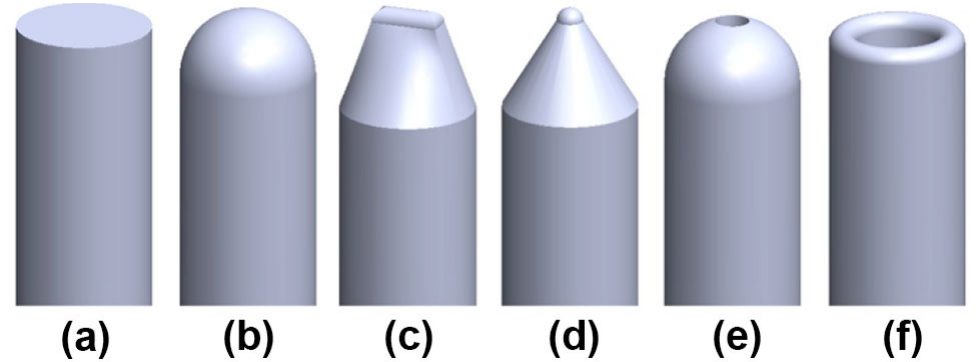
Indenter tip shapes. From left to right: (a) stamp indenter, (b) spherical indenter, (c) wedge indenter, (d) pointed indenter, (e), hollow spherical indenter with guidewire passage, and (f) ringed indenter.

To explore the potential of dynamic impulse for puncturing a CTO and to allow for testing multiple tip shapes, the indenter tip was designed to be interchangeable. For the tip shapes, inspiration was drawn from commonly used indenter shapes in the field of rock fracturing and needle interventions. In total, six tip shapes were chosen (Fig. 3): two blunt solid shapes (a right-angled *stamp* indenter and a *spherical* indenter with the tip radius equal to the indenter radius), two sharp solid shapes (a *wedge* indenter and a *pointed* indenter, both with an edge radius of 0.2 mm), and two hollow shapes (a *hollow spherical* indenter and a *ringed* indenter). The hollow-shaped indenters allow for guiding another system through the shaft and tip, such as a guidewire or balloon catheter, in a future (hollow) prototype, which could be advantageous for clinical use. The *ringed* indenter was designed with a maximized inner passage diameter: the indenter diameter minus four times the edge radius. The *hollow spherical* indenter was designed with a spherical tip and a Ø0.4 mm lumen, through which the thinnest (coronary) guidewires can pass.

#### Indenter Stop

In order to make sure that the indenter has a controllable reach, a stop mechanism was integrated into the design. The stop mechanism is connected to the indenter by means of a rod (Ø1 mm) and is positioned proximal to the position block (Fig. 2). During spring loading, the indenter stop, and thus the indenter, is pulled backwards (i.e., towards the handle of the instrument) until maximum spring compression is reached. Subsequently, the indenter stop is released and accelerated forward by the spring force until it reaches the position block.

### Reload Mechanism

To allow for multiple impacts onto the CTO, a reload mechanism was implemented in the prototype. To reload the device, we used a compliant lock mechanism consisting of a gripper with two flexible barbed plates (Fig. 2, yellow) and an inner movable core (Fig. 2, blue). By translating the gripper forward into the hollow section of the indenter stop, the plates of the gripper deform inwards and passively lock into place. Subsequently, the core is pushed forward into the hollow section to actively lock the grip. We opted for a mechanism that works without control feedback, since most of the haptic feedback will be lost during operation.

### Prototype Tip Design

**Figure 4:**
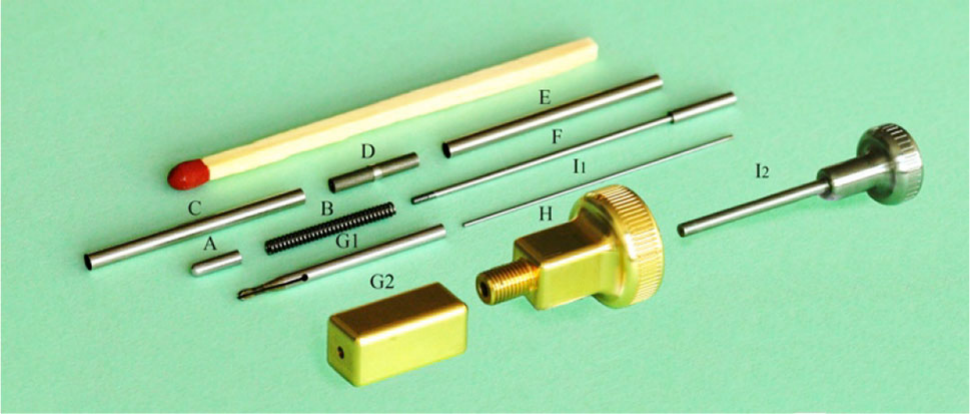
Prototype tip design and control interface. Letter indications: (A) Indenter, (B) Compression spring, (C) Capillary tube front, (D) Position block, (E) Capillary tube back, (F) Indenter stop, (G1) Gripper reload mechanism, (G2) Square sliding bearing connected to the gripper, (H) Handgrip gripper, (I1) Core reload mechanism, and (I2) Handgrip core. The match is shown for scale purposes.

**Figure 5:**
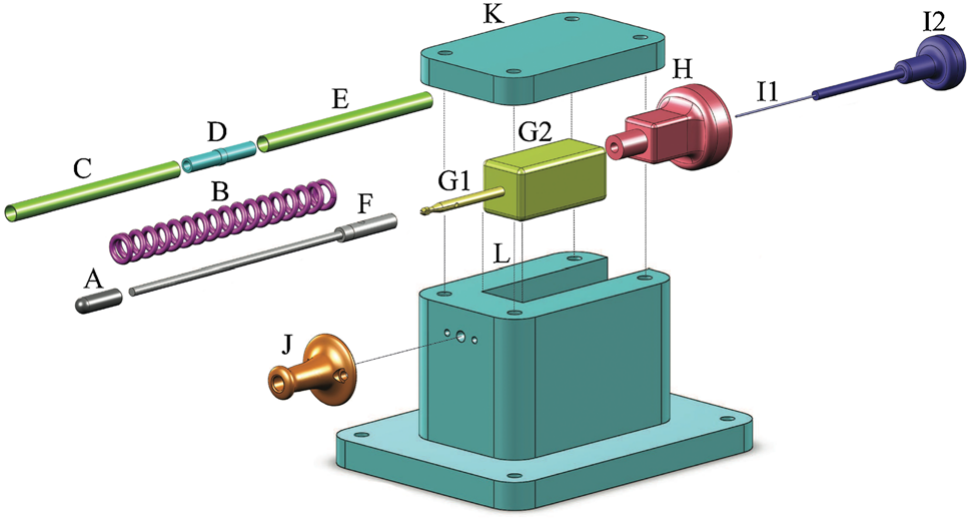
Final design. Letter indications: (A) Indenter, (B) Compression spring, (C) Capillary tube front, (D) Position block, (E) Capillary tube back, (F) Indenter stop, (G1) Gripper reload mechanism connected to (G2) Square sliding bearing, (H) Handgrip gripper connected to G2 (and thus the gripper G1) by means of a screw thread, (I1) Core reload mechanism connected to (I2) Handgrip core, (J) Support capillary tube, (K) Lid, and (L) Enveloping box. In the bottom of part L, a slot is present through which a screw is inserted (not indicated) and connected to G2 to limit the reach of the gripper (G1; Fig. 4). G1, G2, and H are inserted in the rectangular cutout of the enveloping box L.

 A functional prototype was manufactured based on the final tip design (see Figs. 4, 5). We decided to design a rigid prototype in which the shaft is scalable to different lengths and allows for future redesign into a flexible prototype. The outer shaft of the prototype tip is subdivided into three parts to improve manufacturability and consists of two standardized Ø2.0 × 0.1 mm (Ø × wall thickness) stainless steel (*SS*) capillary tubes (C and E in Fig. 4) that fit around both ends of the position block (containing a Ø1.2 mm central lumen; D in Fig. 4; *SS*). The compression spring (B in Fig. 5; *C00170003016,* Associated Spring SPEC, Evasham, UK; *Ø*_*outer*_ = 1.7 mm; *Ø*_*wire*_ = 0.32 mm, *L* = 16 mm; *K* = 1.3 N/mm; *L*_*max*_ _*compression*_ = 3.5 mm) is placed distally to the position block within the capillary tube. The indenter consists of two interconnected (screwable) parts: (1) the indenter tip (A in Fig. 4; Ø1.8 mm; *L* = 6 mm; *SS*) with variable tip shapes, and (2) a Ø1.0 mm rod connected to the indenter stop (F in Fig. 4; Ø1.8 mm; *SS*). The tip of the gripper (Ø1.8 mm; Ø0.5 mm inner lumen; *SS*; G1 in Fig. 5) fits within the hollow section (containing of a narrowed opening and a 45° slanted surface) of the indenter stop (distal end of F in Fig. 4), whereas the other (proximal) end of the gripper is connected to a square sliding bearing (G2 in Fig. 4; brass) that is designed to fit in the handle. The compliant movement of the gripper, necessary to allow the gripper move through the narrowed opening of the hollow part, is possible by the axial slit (*w* = 0.35 mm). The locking core (I1 in Fig. 4; Ø0.5 mm; tempered steel) runs through the gripper towards the handle where it is connected to a square sliding bearing (G2 in Fig. 4).

### Handle Design

**Figure 6:**
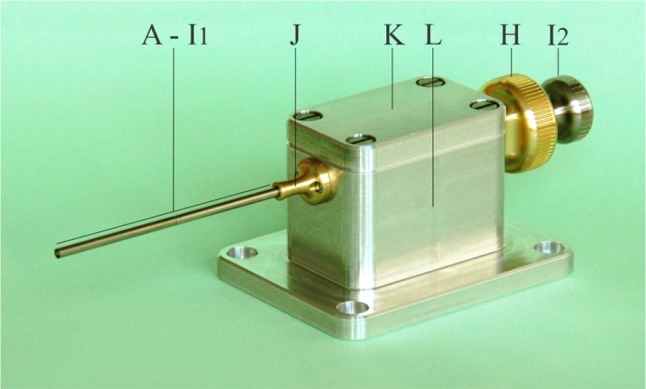
The complete assembled prototype. Letter indications: for (A–I2) see Fig. 4, (J) Support capillary tube, (K) Lid, and (L) Enveloping box. Part G (not indicated, see Fig. 4) is placed inside part L. In the bottom of part L a screw is inserted (not indicated) to limit the reach of the gripper (G1; Fig. 4).

The handle (Figs. 5, 6) mechanically controls the gripper and core at precise distances and allows for locking the mechanism in the “loaded” position. The main enveloping box [26 mm × 41 mm × 25 mm (*l* × *w* × *h*)] consists of a bottom (L in Figs. 5, 6) and a top part (K in Figs. 5, 6), both made of aluminum, and functions as the handle of the device, allowing for loading, locking, and surmounting of the prototype in an experimental setup (Fig. 6). An axial slot was carved in the bottom of the square sliding bearing (connected to the gripper; G2 in Fig. 5), which, together with a screw in the enveloping box, limits the motion range of the gripper. The gripper is controlled by the square sliding bearing (G2 in Fig. 5) that is connected to a handgrip (H in Fig. 5; brass) by means of a M6 screw thread. The core is connected to a second handgrip (I2 in Fig. 5; *SS*) that is guided through the brass handgrip connected to the gripper. Finally, a support part (J in Figs. 5, 6; brass) is connected to the proximal end of the box and guided around the capillary tube to prevent buckling and bending of this structure.

**Figure 7:**
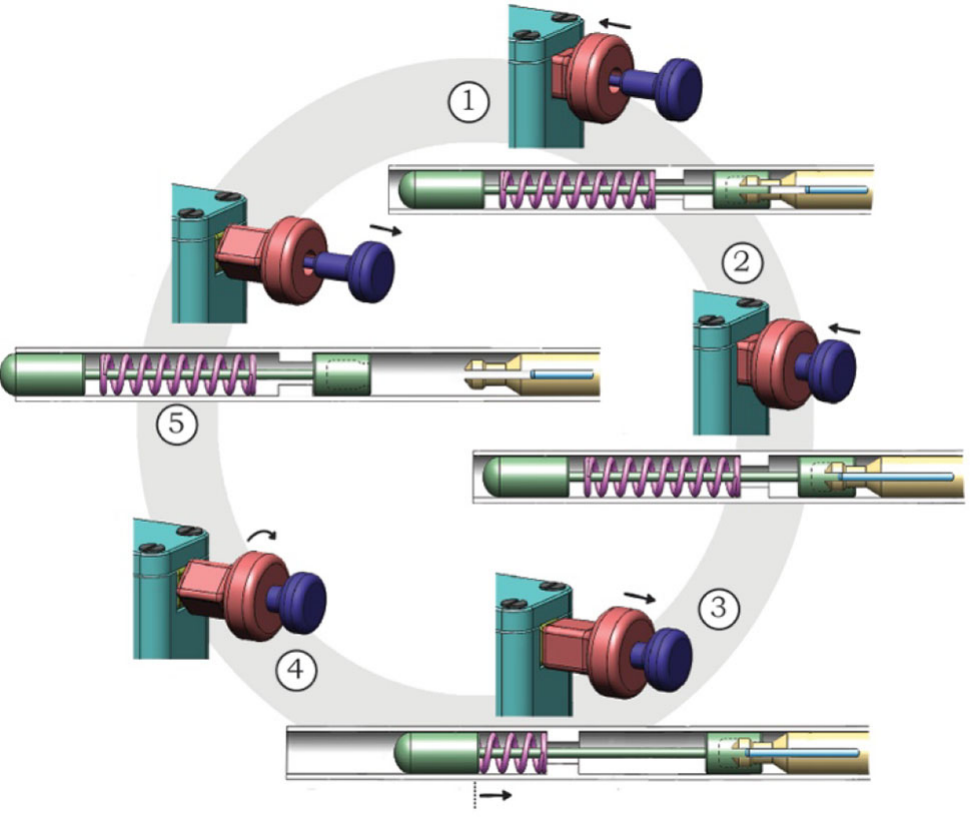
Working principle of the handle. (0) The gripper is decoupled from the system (neutral position). (1) The control input gripper (red: H), and thus the gripper (yellow: G1), is translated forward to couple the gripper (yellow: G1) with the indenter (A). (2) The gripper (yellow: G1) is locked in place by translating the control input core (blue: I2), and thus the core (I1), forward. (3) The control input gripper (red: H), and with it the control input core (blue: I2), is pulled backwards to load the prototype (loaded position). (4) If desired, the prototype can be locked in the loaded position by rotating the control input gripper (red: H). (5) The prototype is released by pulling the locking core (blue: I2) further backwards.

An overview of the steps to load, trigger, and release the indenter is given in Fig. 7. (0) Before loading the spring, the control input core (I2 in Fig. 5) is pulled approximately 10 mm out of the handgrip. (1) The handgrip is pushed into the handle until the gripper is positioned inside the hollow insert of the indenter (connected position in Fig. 2). (2) The handgrip of the core (I2 in Fig. 5, 6) is pushed into the handle to lock the gripper in place (locked position in Fig. 2). (3) Both the handgrip and control input core are pulled out of the handle until the connection between the handgrip and the square sliding bearing becomes visible (G2 and H in Fig. 5; loaded position in Fig. 5). (4) At this stage, the gripper and indenter can be locked in the “loaded” position (or the spring compression distance can be altered) by twisting the handgrip (H in Figs. 5, 6). (5) To release the spring, the core handgrip (I2 in Figs. 4, 5) is pulled out of the handle, releasing the gripper and allowing the indenter to accelerate (towards the ultimate position in Fig. 2).

## Materials and Methods

### Experiment 1: Mechanical Performance of the Prototype

In Experiment 1, the indenter momentum and the impact peak force were measured as a function of the strike distance, the spring compression distance, and the medium through which the indenter was launched.

#### Measurement Variables

##### Dependent Variables

1. *The indenter momentum (p)* generated by the compression spring. *p* was calculated by multiplying the indenter mass (*m*) with the indenter velocity (*v*).
2. *The impact peak force (F*_*peak*_ *)* delivered by the indenter.

##### Independent Variables

1. *Strike distance* To evaluate the effect of the distance between the prototype tip and the CTO (e.g., due to incorrect positioning) on the indenter momentum and impact peak force, two strike distances (i.e., the distance from the indenter in neutral position [see Fig. 2] to the object onto which the impulse is delivered) were tested: 1 mm and 3 mm. The former was selected as the minimum distance between the indenter and the target object, as this allowed the indenter to bounce back after the collision. By bouncing back, static loading of the CTO was prevented. The 3 mm distance was just under the maximum indenter reach (designed at 4 mm).
2. *Spring compression distance* To evaluate the effect of the actuation force on the indenter momentum, three spring compression distances were tested: 3.5 mm (corresponding to the maximum compression distance of the spring), 2.8 mm, and 2.0 mm. The 2.8 mm and 2.0 mm spring compression distances were realized by twisting (and thus unscrewing) the brass handgrip H (Figs. 4, 5) 360^o^ and 720^o^, respectively. The 2.0 mm spring compression distance corresponded to the minimum distance required for the indenter to reach an object at 3 mm distance.
3. *Surrounding medium* The mechanical performance was evaluated in air and in Blood-Mimicking Fluid (BMF), with the latter approximating the clinical environment more closely than air. The BMF was made of 25% weight percentage (wt) glycerine and 75 wt% clear water, a mixture widely used to simulate blood (sometimes with added element such as nylon particles and sodium iodide to improve the optical properties for Doppler flow and particle image velocimetry measurements).^18^–^21^ In Cheng *et al*.,^21^ an exponential formula was presented to calculate the viscosity of a glycerol-water mixture. The formula was numerically derived based on experimental data from earlier studies and was used to develop and validate a correlation model that estimates the viscosity of glycerol-water mixtures. The formula applies for all glycerol concentration (0 and 100 wt%) and temperatures between 0 and 100 °C. According to this formula, the viscosity of blood (2.5–2.8 mPas at 36–40 °C) at an ambient temperature of 18 °C can be approximated with a mixture of 25 wt% glycerine and 75 wt% clear water.

#### Measurement Setup

**Figure 8:**
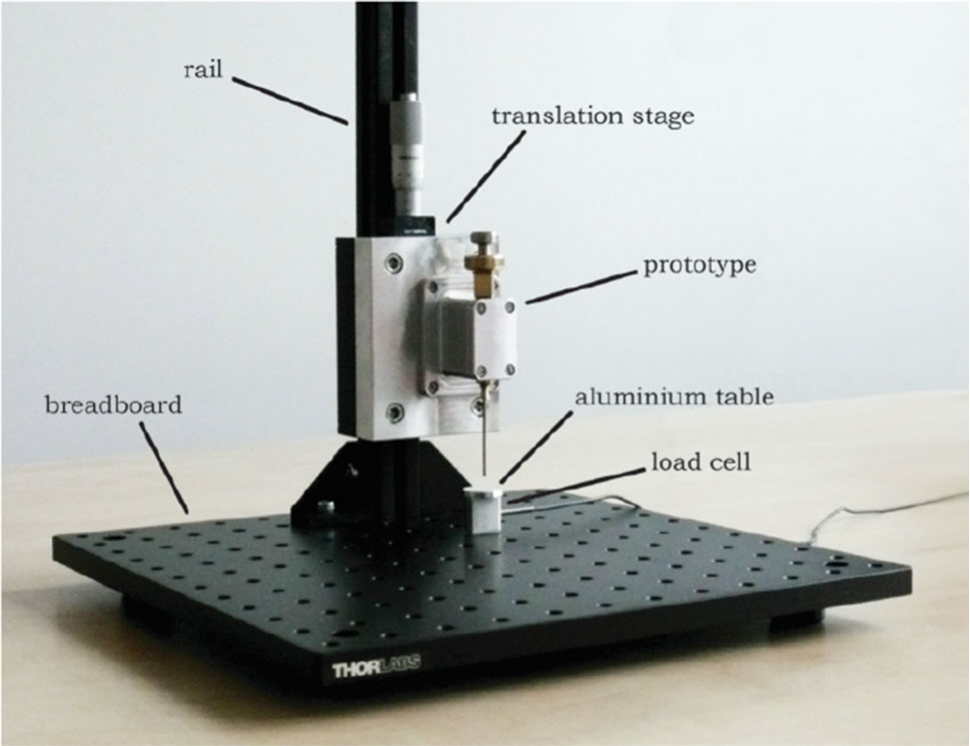
Measurement setup for Experiment 1. The measurement setup consisted of a breadboard, (25 mm construction) rail, and a single-axis translation stage to which the prototype was connected. The prototype tip was pointing downwards onto a miniature load cell, on top of which a small aluminum table or bucket was fastened to receive the impact peak force in air and blood mimicking fluid, respectively. For the velocity measurement, a HSV-camera was placed next to the breadboard.

The prototype was suspended vertically, with its tip pointing downwards, in a construction of Thorlabs optomechanics (Thorlabs, Inc., Newton, NJ), consisting of a solid aluminum breadboard (*MB3030/M*), a construction rail (*XE25L375/M*), and a single-axis translation stage (*PT1/M*) (Fig. 8). The prototype was fastened on the translation stage using an intermediate plate construction. The indenter strike was received by a small aluminum table (for the measurements in air) or bucket (in which the prototype tip was immersed for the measurements in BMF) fastened on top of a miniature load cell (*LSB200, FSH00103*, FUTEK Advanced Sensor Technology, Inc., Irvine, CA; see Fig. 8). The effect of gravity in this experiment was considered negligible, since the estimated gravity force is three orders of magnitude smaller than the maximum spring force.

For the determination of the indenter momentum, the indenter velocity was measured from High Speed Videos (HSVs) made with a *Photron Fastcam APX*-*RS* (Photron, Inc., San Diego, CA) at a frame rate of 10 kHz, and the indenter mass was measured with a high precision balance (*Mettler PJ360 DeltaRange*, Mettler-Toledo International Inc., Columbus, OH).

The impact peak force was measured with the miniature load cell, connected to an analogue signal conditioner (*CPJ RAIL*, SCAIME, Annemasse, France) and a data acquisition system with a sampling rate set to 50 kHz (*NI USB*-*6211*, National Instruments Corporation, Austin, TX). This system was controlled through LabVIEW 2014 (National Instruments Corporation, Austin, TX). For each of the impact peak force measurements, 1 s of data before and 1 s after the indenter strike were sampled.

#### Measurement Protocol

The impact peak force was measured for all 12 conditions defined by the three independent variables: 2 strike distances × 3 spring compression distances × 2 surrounding media. The indenter velocity was measured only in air, thus for 6 conditions (2 strike distances × 3 spring compression distances), as it was not possible to video capture the motion in BMF. Each condition was tested five times.

#### Data Analysis

Per measurement, the indenter velocity was determined by dividing the travelled distance of the indenter tip (from neutral position to impact) with the elapsed time (0.1 ms per image) using the associated software package Photron FASTCAM Viewer v3.5.3. Subsequently, the indenter moment per measurement was determined by multiplying the indenter velocity with the indenter mass. Finally, per condition, the mean indenter velocity and mean indenter momentum with their associating standard deviations were determined across the five repetitions.

The data from the miniature load cell was processed with MATLAB 2013b (The Mathworks, Inc., Natick, MA) to identify the impact peak force (highest value measured) for each measurement and the mean impact peak force (i.e., the mean of the five impact peak forces measured) per condition. Furthermore, to determine the effect of the surrounding medium on the impact peak force, the ratio between the mean peak force in BMF was compared to that in air (*F*_*peak*_ BMF*/F*_*peak*_ Air [-]) for each condition.

### Experiment 2: Puncture Performance of the Prototype

In Experiment 2, the puncture performance of the prototype was evaluated on a CTO model by measuring the number of punctures, the puncture success rate, the number of indenter strikes necessary to achieve a puncture, and the distribution of energy dissipation between the indenter and the CTO model, as a function of the indenter tip shape and the hardness of the CTO model.

#### Measurement Variables

##### Dependent Variables

A CTO is a heterogeneous mix of materials, including intracellular and extracellular lipids, smooth muscle cells, a collagen-rich extracellular matrix, cholesterol, dense collagen, and calcium.^1^,^17^ Collagen forms the major structural component of the extracellular matrix of the CTO. CTOs can be soft, hard, or contain both hard and soft regions.^1^ Soft CTOs are usually younger than hard CTOs and mainly consist of fat-laden cells and loose fibrous tissue, whereas hard CTOs are characterized by dense fibrous tissue and contain calcified regions.^1^ This age-related increase in calcium and collagen content of CTOs substantiates the difficulty of crossing older occlusions.

The calcification process is similar to bone development and generally consists of two main mechanisms.^17^ (1) High phosphate levels in the CTO cause smooth muscle cells to differentiate into osteoblasts, eventually resulting in the formation of HydroxyApatite (HA; a calcium phosphate usually denoted as Ca_10_(OH)_2_(PO_4_)_6_)) crystals and thus calcified regions.^21^ (2) Cytokines (i.e., proteins that can signal other cells) signalize osteoblast and osteoblast-like cells to the CTO from circulating stem cells, smooth muscle cells, or pericytes (i.e., contractile cells that wrap around the endothelium of the blood vessel wall), which will eventually develop into HA crystals and thus calcification.^21^

Along the length of the CTO, three regions can be distinguished, with distinct material properties: (1) the proximal cap, (2) the main body (core), and (3) the distal cap of the CTO.^17^ The proximal cap is a thickened fibrous structure at the proximal end (i.e., the first side encountered by the blood flow) of the CTO. It is the hardest part of the lesion and contains particularly densely-packed collagen and calcified tissue. The distal cap is also a thickened structure but somewhat thinner and softer than the proximal cap. The core between the two caps is softer and consists mostly of organized thrombus and lipids.^1^

In order to evaluate the performance of the prototype to cross a CTO, we decided to focus on puncturing the proximal cap of heavily calcified CTOs, as this is the hardest, most difficult part to penetrate during PCI. Puncture performance was evaluated in terms of efficiency and efficacy. Specifically, the following three measures were defined:

1. *Number of punctures* This measure was used as an indication of the efficacy of the puncture (binary classification: puncture vs. no puncture). In our experiment, puncture is defined as complete breakage of the proximal cap. The maximum number of strikes allowed per trial was set to 10.
2. *Number of strikes for puncture* This measure was used as an indication of the efficiency of the puncture (scale classification).
3. *Energy transfer from the indenter to the CTO model* The main working principle of the impact method is the collision between a moving body (the indenter) and an initially non-moving body (the CTO). During impact, kinetic energy of the indenter can be transferred into kinetic energy of the CTO or dissipated into heat, deformation, or fracture. There are two main types of collisions that can occur: (1) an elastic collision and (2) an inelastic collision. An elastic collision is featured by conservation of kinetic energy, whereas in the case of an inelastic collision kinetic energy dissipates into other forms of energy. For our application, the elastic collision is drawn as the *“worst case scenario”*, as both the indenter momentum and kinetic energy are conserved and transferred to the CTO, leading to CTO displacement. The *“ideal scenario”*, on the other hand, is a perfect inelastic collision, where the two colliding bodies stick together after collision, and the maximum amount of kinetic energy is absorbed by the CTO, potentially resulting in CTO puncture. Whether the occurring collision is elastic or inelastic depends on the characteristics of the CTO and its environment, such as the CTO mass, damping coefficient, and spring constant, as well as on the velocity and mass of the indenter. To determine the type of collision, the following two variables were measured:
*Indenter bounce velocity* To estimate the amount of energy absorbed by the environment and the CTO combined and to compare this energy absorption in the cases of puncture versus no puncture, the average velocity of the indenter after bouncing away from the proximal cap was measured.
*CTO displacement* To determine the type of collision (i.e., elastic or inelastic) and estimate the amount of energy absorbed by the environment and CTO separately, the CTO displacement was measured.

All measurements in Experiment 2 were conducted with the maximized impact condition as identified from Experiment 1 (i.e., strike distance of 1 mm and spring compression distance of 3.5 mm). A pilot study we conducted indicated that testing with lower impact forces would only provide trivial evidence of a lower efficacy and efficiency than the maximized condition.

##### Independent Variables

The puncture performance of the prototype was evaluated as a function of the following two variables:

1. *Indenter tip shape* The tip shape that is the most efficient and effective in puncturing a CTO depends on the material properties of the CTO. Fracture of brittle materials leads to material pulverization and chip formation and is usually conducted with blunt tip shapes. Fracture of ductile materials, on the other hand, associates with material tearing and is commonly achieved by using sharp tip shapes, such as needles. Due to the uncertainty of the material properties of the CTO and its environment, the previously described tip shapes were tested: *stamp*, *spherical*, *wedge*, *pointed*, *hollow spherical with guidewire passage*, and *ringed*.
2. *Proximal cap hardness* The development of a representative CTO animal model has been proven difficult mainly due to the lack of spontaneous atherosclerosis in animals.^1^ Recently, Suzuki *et al*.^22^ succeeded in developing an animal model (pig and rabbit) that was histologically similar to human CTOs by implanting hydroxyapatite-coated bio-absorbable polymer sponges in the coronaries. Even though the use of animal models is preferred for device evaluation, these are expensive and do not allow for consecutive tests and evaluation under constant test conditions. Therefore, it was decided to build an artificial CTO model to determine the feasibility of technology. The proximal cap model was made from calcium sulfate (CaSO_4_; *SHERAALPIN Hartgips hellblau*, SHERA Werkstoff-Technologie GmbH & Co. KG) and a gelatin mixture (*sheet gelatin*, Dr. Oetker, Bielefeld, Germany). These materials were selected to mimic the high calcium and collagen concentrations found in the proximal caps of heavily calcified CTOs. Calcium sulfate is a mineral that can be found in the human body and has similar mechanical properties as organic HA crystals. Specifically, the compressive strength of CaSO_4_ is 44 MPa (after 1 h) and that of (carbonated) HA formed at body temperature approximately 30–70 MPa.^23^ Higher compressive strengths are found in calcium-deficient HA (Ca_9_HOH(PO_4_)_6_ (approximately 170 MPa) and sintered HA (up to 500 MPa).^23^ However, the HA crystals found in CTOs are most often referred to as carbonated HA; it is unclear whether calcium-deficient HA is present in CTOs and whether sintered HA is formed in a different way than natural HA (as it is manufactured using significantly higher temperatures than body temperature). Therefore, the lower compressive strength values found for carbonated HA seem more likely to represent naturally found HA in CTOs.
  To mimic the densely-packed collagen and overall heterogeneous morphology of the CTO at the proximal cap, the calcium sulfate was mixed with gelatin, which is the product of structural and chemical degradation of collagen.
  Transluminal calcium ≥50% (as assessed by Multi-Detector Computed Tomography (MDCT)) is a strong predictor for PCI failure. In a study of Cho *et al*.,^24^ the calcium burden of CTOs was determined using MDCT. In this study a mean calcium percentage (determined as the percentage of calcium cross-sectional area divided by vessel area) of 53.9 ± 20.3% was found in the PCI-failure cases. As we focus on crossing the most calcified CTOs, proximal caps with three degrees of hardness were tested (called henceforth *ductile*, *reference*, and *brittle*) with a calcium percentage over 50%. The *ductile* model was created with 50 wt% calcium powder and 50 wt% gelatin mixture in a Ø10 mm circular slice and represented the mean calcium percentage in CTOs in the PCI-failure group in Cho *et al.* 24 The *reference* model was created with 75 wt% calcium powder and 25 wt% liquid gelatin mixture. Whereas reported average concentrations of calcium content within calcified CTOs are generally lower than 75%,^24^ CTO caps are heterogeneous and thus local calcium concentrations as high as 75% are expected. Next to the reference model, a *brittle* model (77 wt% calcium powder and 23 wt% clear water) was also prepared to represent the most heavily calcified CTOs that can be encountered.
  Proximal caps are on average 0.5 mm thick.^25^ Due to molding difficulties to produce homogeneous 0.5 mm models, a model thickness of 1 mm was set.

**Figure 9:**
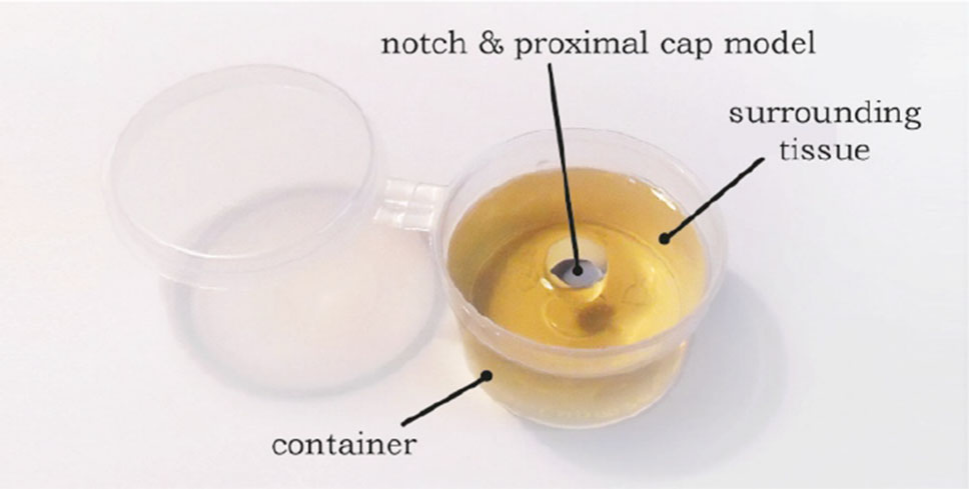
The CTO model. The CTO model consisted of a small container (Ø50 mm, 26 mL) filled with gelatin representing the surrounding cardiac tissue (environment model), in which a Ø1 mm, 1 mm thick slice of stiffened plaster cast mixed with gelatin/water was placed.

Unlike acute occlusions, CTOs are strongly connected to the blood vessel wall, which in turn is connected to cardiac muscle tissue of the heart. In order to simulate the environment of the CTO while allowing for consecutive measurement of the prototype under stable test conditions, it was chosen to simulate the environment of the CTO using a mixture of 25 wt% gelatin and 75 wt% water. When set, this mixture has an estimated Young’s modulus between 100 and 130 kPa, resembling the Young’s modulus of cardiac muscle tissue.^26^ Furthermore, to emulate the strong blood vessel wall connection, a Ø10 mm notch with approximately 7 mm depth was created in the center of the gelatin mixture (Ø50 mm, 26 mL; Fig. 9) in which the proximal cap model was placed. This notch also allowed for simultaneous stretching and compressing of the gelatin, similar to the clinical situation. For a complete overview of the steps followed to manufacture the CTO models, see the Appendix.

#### Measurement Setup

**Figure 10:**
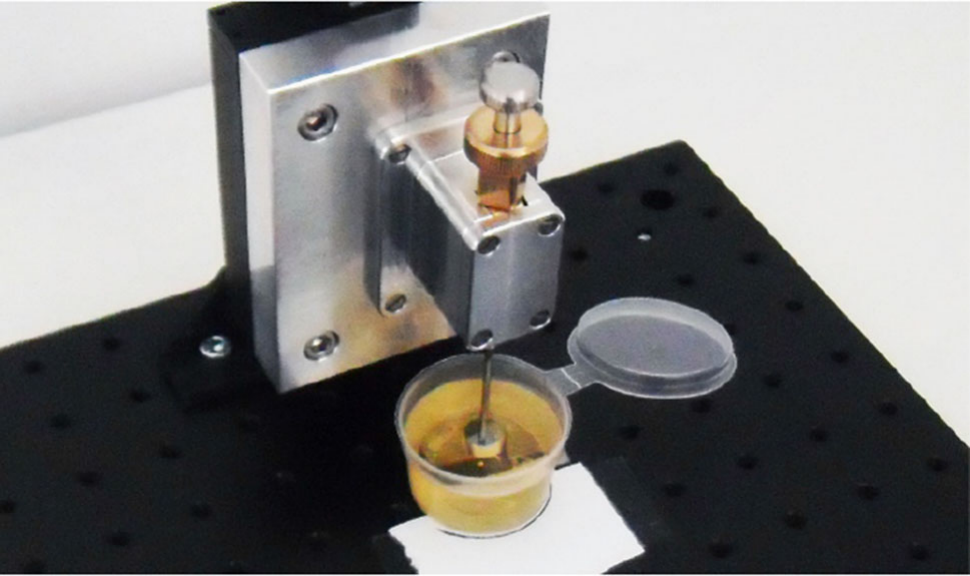
Measurement setup for Experiment 2. The measurement setup consisted of the same Thorlabs facility as in Experiment 1 (see Fig. 10), with the only difference that instead of the load cell, the CTO model (see Fig. 11) was placed directly under the prototype tip.

For Experiment 2, the prototype was suspended in the same way as described in Experiment 1 (see Fig. 10). The sole difference was that, instead of load cell, a CTO model was placed directly under the prototype (Fig. 9).

#### Measurement Protocol

The number of punctures and the number of strikes were measured for all 18 conditions defined by the two independent variables (6 indenter tip shapes x 3 degrees of model hardness). Additionally, to evaluate the overall effectiveness of the impact method as a function of the CTO model hardness, the results from all six tip shapes were grouped together per CTO model, resulting in a data analysis of 3 groups within the 18 performed trials. Each of the *ductile*, *reference*, and *brittle* models were prepared in a single batch to limit the variability in the CTO models, and all measurements were conducted within 4 h, to limit the effect of CTO model hardening over time. Three measurements were conducted per condition.

The measurements of the energy transfer from the indenter to the CTO model were conducted with the spherical tip shape. One energy transfer measurement was conducted for the *ductile* and *reference* models and two for the *brittle* model.

#### Data Analysis

Per indenter tip shape and CTO model, the number of strikes to achieve puncture and the number of punctures were counted. Furthermore, per CTO model (tip shapes grouped together), the number of punctures, the success rate (defined as the percentage of punctures across the 18 conditions), as well as the minimum, maximum, and mean number of strikes across the 18 conditions to achieve puncture were determined.

To estimate energy transfer from the indenter to the CTO, the indenter momentum and kinetic energy ($E_{kinetic} = \frac{1}{2}mv^{2}$, with *m* = mass of the indenter [kg] and *v* = the velocity of the indenter [m/s]) 0.1 ms before and 0.1 ms after impact was calculated using the indenter velocity derived from the HSV images using the method previously described. Furthermore, the maximum displacement of the CTO model per measurement was manually derived from the captured HSV images, and the energy absorbed by the (gelatine) environment was calculated as $U = \frac{1}{2}VE\varepsilon^{2}$, with *V* = volume of the gelatine (26 mL), *E* = Young’s modulus of the gelatine (115 kPa), and *ε* = strain of the gelatine during impact (approximated as the displacement of the proximal cap model divided by the original location).

### Control Experiment

In order to compare the performance of the newly developed device with that of currently used guidewires and to assess the validity of the used CTO model, a separate control experiment was performed. In this experiment, a rigid Ø0.4 mm stainless steel rod was used to model the flexible guidewire tip. The rigid rod model was driven into the ductile and reference CTO model (equal to the previously described models) by a linear stage. The force required to puncture the CTO model as well as the resulting displacement of the model were measured. The velocity of the insertion ranged between 0.1, 0.5, 5, 50, 500, and 1000 mm/s, to simulate low velocity and high velocity insertion. Currently used insertion velocities of the guidewire are not described in literature. Therefore, a wide velocity range was tested, with the higher insertion velocities chosen to determine the effect of high-speed insertion.

## Results

### Experiment 1: Mechanical Performance of the Prototype

#### Indenter Momentum

**Figure 11:**
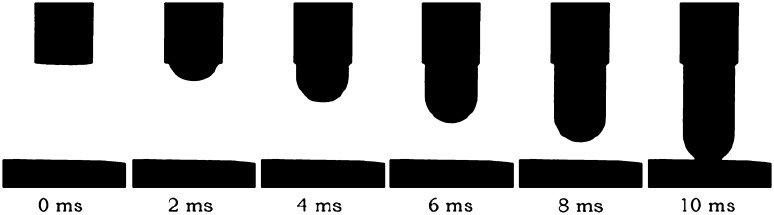
Example of an HSV image sequence of an indenter strike with an object distance of 3 mm and a spring compression distance of 3.5 mm. An average indenter velocity of 3.0 m/s can be derived.

In Fig. 11, a typical HSV image sequence of an indenter strike is illustrated. The calculated momenta for each of the six tested conditions (2 strike distances × 3 spring compression distances) are illustrated in Table 1. The mean velocity generated with maximized actuation force was 3.4 m/s, which translates to a mean indenter momentum of 1.33 mNs (for *m* = 0.39 grams). Decreasing the compression spring distance and increasing the strike distance was associated with a decrease in indenter velocity. In the condition of minimal spring compression distance (2 mm), the indenter did not reach the ultimate position (3 mm).

**Table 1:** Velocity (mean ± standard deviation, *n* = 5) and mean indenter momentum (*p* = *mv*) for the six conditions.

Strike distance/spring compression distance	Indenter velocity (*v;* m/s)	Indenter momentum (*p;* mNs)
1 mm/3.5 mm	3.4 ± 0.11	1.33 ± 0.04
3 mm/3.5 mm	3.0 ± 0.16	1.17 ± 0.06
1 mm/2.8 mm	2.2 ± 0.07	0.86 ± 0.03
3 mm/2.8 mm	1.3 ± 0.20	0.51 ± 0.08
1 mm/2.0 mm	1.0 ± 0.05	0.39 ± 0.02
3 mm/2.0 mm^a^	–	–

^a^For this condition, the velocity of the indenter could not be determined with accuracy due to the minimal travelled distance of the indenter out of the outer shaft

#### Impact Peak Force

##### Impact Peak Force in Air

**Figure 12:**
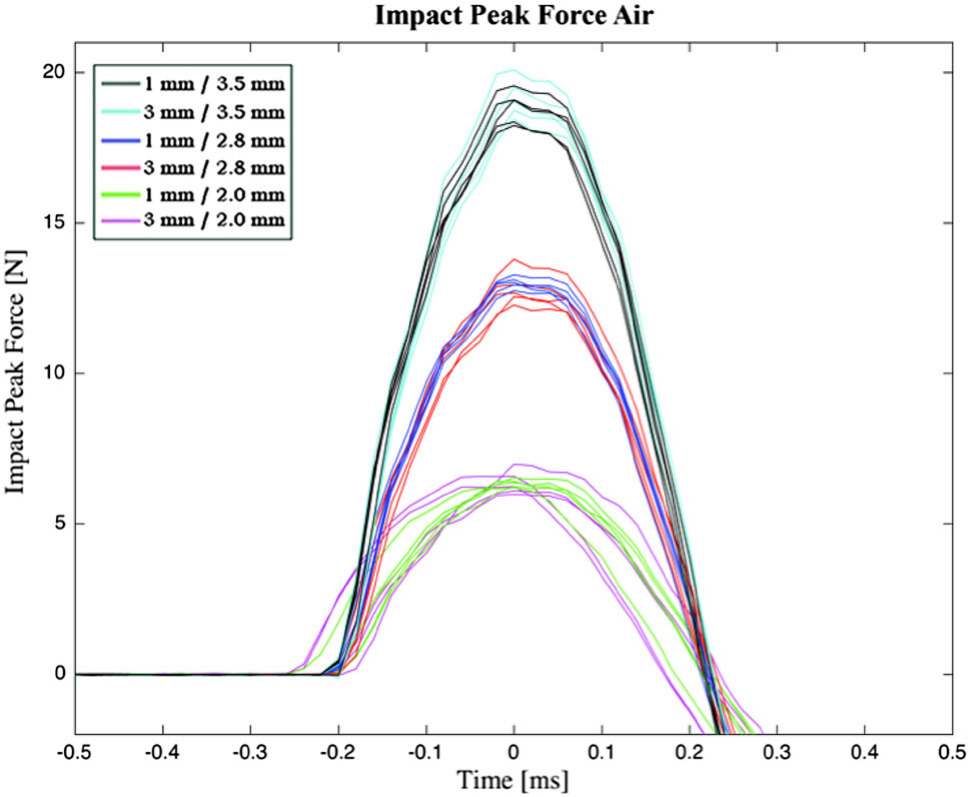
Impact peak force of the indenter in air. Each color (*n* = 5 strikes) represents a different strike distance x spring compression distance combination. For each indenter strike, represented by one line, time 0 corresponds to the maximum impact peak force.

Figure 12 shows the impact peak force measurements in air. The data were approximately normal (based on a one-sample Kolmogorov–Smirnov test). The impact peak forces (mean ± standard deviation, *n* = 5) are presented in Table 2. The maximum mean impact peak force was approximately 19.2 N, generated with maximized actuation force and independent of the strike distance. The impact peak force decreased with decreasing spring compression distance.

**Table 2:** Impact peak force *F*_*peak*_ (mean ± standard deviation, *n* = 5) in air and in blood mimicking fluid (BMF) for the six conditions, and ratio of the impact peak force in BMF and air (*F*_*peak*_ BMF*/F*_*peak*_ air).

Strike distance/spring compression distance	*F*_*peak*_ air (N)	*F*_*peak*_ BMF (N)	*F*_*peak*_ BMF*/F*_*peak*_ air (-)
1 /3.5 mm	18.9 ± 0.55	9.2 ± 0.08	0.49
3 /3.5 mm	19.2 ± 0.65	7.9 ± 0.75	0.41
1 /2.8 mm	13.0 ± 0.20	5.8 ± 0.29	0.45
3 /2.8 mm	12.9 ± 0.58	4.1 ± 0.74	0.32
1 /2.0 mm	6.4 ± 0.13	2.4 ± 0.20	0.38
3 /2.0 mm	6.4 ± 0.41	0.7 ± 0.68	0.11

##### Impact Peak Force in BMF

**Figure 13:**
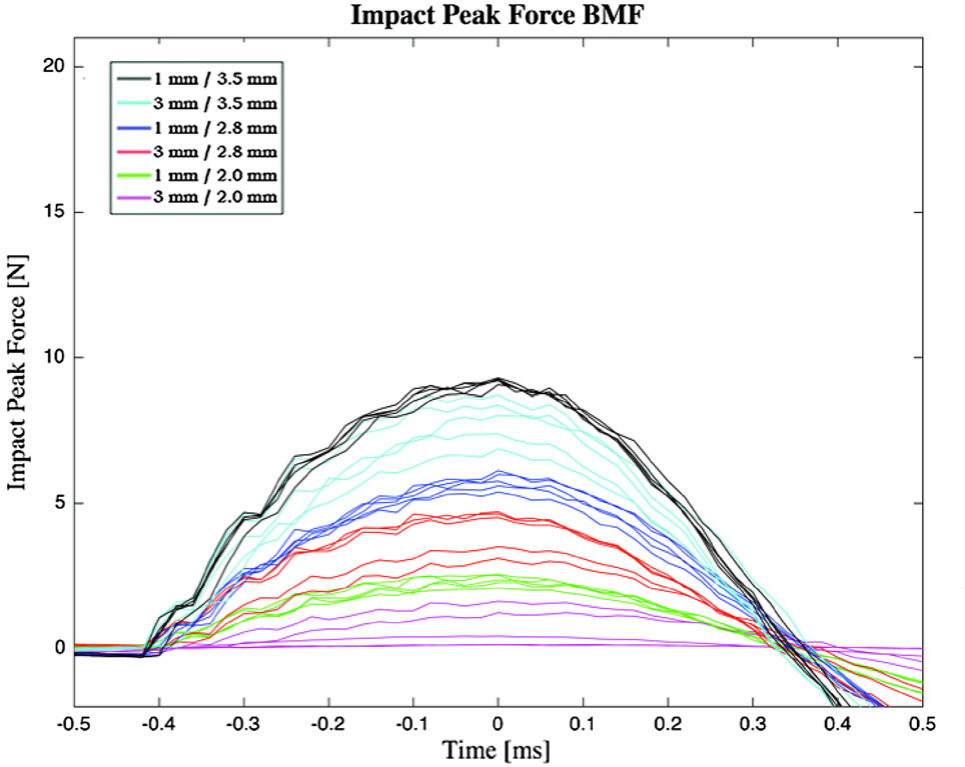
Impact peak force of the indenter in BMF. Each color (*n* = 5 strikes) represents a different strike distance x spring compression distance combination. For each indenter strike, represented by one line, time 0 corresponds to the maximum impact peak force.

Figure 13 shows the impact peak force measurements in BMF. Data were approximately normal. The impact peak force in BMF was approximately a factor 0.49–0.11 lower than that of the impact peak force in air (Table 2). The strike distance was of greater influence on the impact peak forces in BMF than in air, which is illustrated by a lower impact peak force in BMF in comparison to air and *F*_*peak*_ BMF*/F*_*peak*_ air ratio for a strike distance of 3 mm.

### Experiment 2: Puncture Performance of the Prototype

#### Number of Punctures, Success Rate, and Number of Strikes

**Figure 14:**
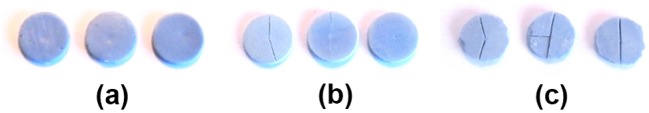
Typical proximal cap models after striking the indenter multiple times onto them. The illustrated proximal cap models were struck with the spherical indenter tip shape, on (A) the *ductile* model (10 strikes on all of the models), (B) the *reference* model (from left to right: 4, 5, and 10 strikes), and (C) the *brittle* model (from left to right: 3, 3, and 2 strikes).

Table 3 shows the puncture performance for the different indenter tip shapes (see also Fig. 14 for a typical result of multiple impact strikes on the three CTO models with the spherical indenter). For the *ductile* model, the indenter left an imprint of plastic material deformation, but did not manage to penetrate the model within 10 strikes. The measurements on the *reference* model did not show convincing results to indicate which tip shapes were the most effective and efficient. However, the *wedge* tip shape was the only tip shape able to puncture the model during all three tests (6 strikes on average). Finally, for the *brittle* model the most efficient and effective tip shapes were the *spherical* (2.7 strikes on average), the *spherical with guidewire passage* (2.7 strikes on average), and the *ringed* (2 strikes on average) tip shape. The *pointed* tip shape was generally less effective and efficient to puncture the *brittle* model, as compared to all other tip shapes. Specifically, the only case in which the *brittle* model was not punctured occurred with the pointed tip shape. Moreover, in its two successful cases, this indenter needed 8 strikes to puncture the model, which is much higher than the average of 2.5 strikes required with the other five tip shapes (*n* = 15).

**Table 3:** Number of strikes to puncture the proximal cap models, using indenters with different tip shapes. The tip shapes are presented in the top row. The CTO model type and test number (*n* = 3) are given in the left two columns.

Type of model	#	Stamp	Spherical	Wedge	Pointed	Spherical GW^a^	Ringed
*Ductile* model	I	No puncture	No puncture	No puncture	No puncture	No puncture	No puncture
	II	No puncture	No puncture	No puncture	No puncture	No puncture	No puncture
	III	No puncture	No puncture	No puncture	No puncture	No puncture	No puncture
*Reference* model	I	9	4	6	No puncture	No puncture	5
	II	No puncture	5	7	7	9	10
	III	6	No puncture	5	No puncture	10	9
*Brittle* model	I	2	3	3	8	3	2
	II	3	3	3	8	2	2
	III	5	2	3	No puncture	3	2

The maximum number of strikes was set to 10, and # indicates the test number (I, II, or III)

^a^Spherical indenter with guidewire passage

Table 4 shows the number of punctures, success rate, and the number of strikes for puncture, for the *ductile*, *reference*, and *brittle* models. It can be seen that the number of punctures (efficacy) and the success rate increased, and number of strikes for puncture (efficiency) decreased with increasing model hardness.

**Table 4:** Number of punctures (all tip shapes combined) for the three CTO models.

Type of model	Number of punctures	Success rate	Mean number of strikes (range)
*Ductile* model	0/18	0%	–
*Reference* model	13/18	72%	7.3 (4–8)
*Brittle* model	17/18	94%	3.4 (2–8)

#### Energy Transfer from the Indenter to the CTO Model

The average velocity of the (spherical) indenter after bouncing from the proximal cap model was approximately 0.5 m/s in the cases in which the proximal cap model was not punctured (Table 5). No differences were observed in the bouncing velocities for the different proximal cap models. A slightly higher bounce velocity of 0.8 m/s was observed when the brittle proximal cap model was punctured (Table 5).

**Table 5:** Energy transfer from the indenter to the CTO model.

Type of model	Initial velocity indenter (m/s)	Initial momentum (mkgm/s)	Initial kinetic energy (mJ)	Bounce velocity indenter (m/s)	Bounce momentum (mkgm/s)	Bounce kinetic energy (mJ)	Loss kinetic energy indenter (mJ)	CTO displace-ment (mm)	Energy absorption gelatin (mJ)	Energy absorption CTO (mJ)
*Ductile* model (no puncture)	3.3	1.29	2.16	0.5	0.20	0.05	2.11 (97.74%)	0.8	0.020 (1%)	2.09 (99.0%)
*Reference* model (no puncture)	3.5	1.37	2.39	0.5	0.20	0.05	2.34 (97.96%)	0.8	0.020 (0.9%)	2.32 (99.1%)
*Brittle* model (no puncture)	3.3	1.29	2.16	0.5	0.20	0.05	2.11 (97.74%)	0.9	0.025 (1.2%)	2.09 (98.8%)
*Brittle* model (punctured)	3.5	1.37	2.39	0.8	0.31	0.12	2.26 (94.78%)	1.4	0.060 (5.4%)	2.20 (94.6%)

**Figure 15:**
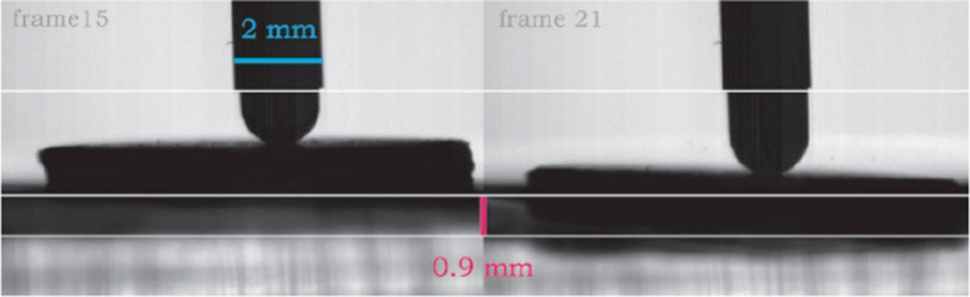
A visualization of the analysis of HSV images to derive the maximum CTO displacement of a non-punctured brittle model using the spherical indenter.

The maximum displacement of the proximal cap models in case no puncture occurred was between 0.8 and 0.9 mm (Fig. 15; Table 5). A larger displacement was observed for the punctured *brittle* model in comparison to the models in which no puncture was achieved, with a maximum of 1.4 mm (Table 5).

Based on this data, the energy loss of the indenter is estimated to be between 2.34 and 2.11 J (~95–98% energy loss, see Table 5). Most of this energy was absorbed by the proximal cap of the CTO model (about 95–99%), whereas the remaining 1–5% was absorbed by the gelatin.

### Control Experiment

The mean force to penetrate through the CTO model was 0.24 ± 0.09 N for the ductile model and 2.7 ± 1.1 N for the reference model. No significant differences were found in penetration force for the different insertion velocities for each CTO model as determined by two separate ANOVAs. The displacement of the CTO model ranged between 1 and 2.7 mm, with the lowest value measured for the highest penetration velocity.

## Discussion

### Summary of Main Findings

#### Mechanical Performance

##### Indenter Momentum

The theoretical maximum velocity of the indenter (frictional effects neglected) was calculated as 6.6 m/s at the moment the indenter protrudes from the prototype, using Eqs. (1–4):

1 $$F = K \cdot x ,$$

2 $$a = \frac{F}{m} ,$$

3 $$v = v_{0} + a \cdot t\;,{\text{ and}}$$

4 $${\text{s}} = s_{0} + v \cdot t,$$

with *a* = acceleration [m/s^2^] of the spring and thus the indenter, *F* = actuation force of the spring [N] of 4.55 N, *K* = spring constant [N/mm] of 1.3 N/mm, *m* = mass of the indenter [grams] of 0.39 grams, *x* = maximum spring compression distance [mm] of 3.5 mm, *s* = distance travelled [mm], *s*_*0*_ = distance travelled at time *t*_*0*_ [mm], *t* = time step [s], *v* = velocity of the indenter [m/s] at *t*_*1*_ = *t*_*0*_ + *t*, and *v*_*0*_ = velocity of the indenter [m/s] at time *t*_0_.

From the indenter mass and theoretical maximum velocity of the indenter, a theoretical maximum indenter momentum of 2.34 mNs can be deducted. The actual (measured) indenter momentum of 1.40 mNs (with an associated velocity of 3.6 m/s) is approximately 40% lower than the theoretical value. It can thus be concluded that friction between the indenter, compression spring, and outer shaft plays an important role within the mechanism. Potentially, a higher efficiency may be realizable by reducing frictional effects. This could, for example, be achieved by a different material choice for the moving parts (the indenter and spring) and the surrounding catheter tube, or by lubricating the moving parts.

##### Impact Peak Force

In Thind *et al*.,^7^ the puncture force of 2, 6, 12, and 15 weeks old rabbit femoral CTOs was measured using a probe similar in profile to a Ø0.36 mm guidewire. These authors found that the puncture force is significantly lower in CTOs younger than 6 weeks of age (0.61 N and 0.78 N in CTOs of 2 and 6 weeks old, respectively) than in those older than 12 weeks (1.21 N and 1.52 N in CTOs of 12 and 15 weeks old, respectively). These values are in line with Roy *et al*.,^27^ in which puncture forces of excised peripheral CTOs between 0.3 and 1.7 N for soft (containing loose fibrous tissue, fat, thrombus, or micro-blood vessels) and hard (containing collagen and speckled calcium) CTOs, respectively, were measured.

In the control experiment, it was also found that the less calcified CTO models required significantly lower penetration forces. For the brittle model slightly higher penetration forces were found compared to the animal model in Thind *et al*.,^7^ which could have been caused by the larger diameter of the rigid rod we used (Ø0.4 mm vs. Ø0.36 mm), the thickness of the proximal cap (1 mm), and the consistency of the model. Nonetheless, the 0.2–2.7 N penetration force found in the artificial CTO model is comparable to the values found in the animal model of Thind *et al*.^7^ and Roy *et al*.^27^

The maximum tip load (i.e., the load in grams that a guidewire can withstand before buckling) of dedicated coronary CTO guidewires ranges between 0.8 and 26.7 grams, which is equal to 0.008 and 0.26 N.^5^ If we compare the maximum tip load of dedicated guidewires (i.e., ~ 0.26 N),^5^ with the required penetration force of the reference CTO model (2.7 N), it becomes clear that buckling is likely when using dedicated coronary CTO guidewires. It can, therefore, be argued that a dedicated CTO guidewire is unable to penetrate the reference model.

In the prototype experiments, maximum mean impact peak forces of 19.2 N and 9.2 N were measured in air and BMF, respectively, which in theory is sufficient to puncture the CTO. Moreover, even though impact peak forces were 50 to 90% lower in the BMF than in air, they were still above the values in Thind *et al*.^7^ It must be noted, however, that since no experiments have been performed on real CTOs, there is still uncertainty about the required impact peak force to puncture real *in vivo* CTOs.

#### Puncture Performance

##### Number of Punctures & Number of Strikes

The highest puncture performance and efficiency was observed for the brittle CTO models. The effectiveness of the impact method to puncture the more ductile CTO models was limited, which can be explained by the fact that ductile materials absorb energy from the impact loads by deformation (elastic and plastic). This finding is consistent with the fact that the impulse method is more widely used to fracture hard and brittle materials, rather than tough and/or elastic materials.

Puncture performance of the prototype was assessed using the six indenter tip shapes on the three proximal cap models. For the *ductile* models, it was expected to see improved puncture performance in terms of number of punctures and number of strikes for puncture with the sharp indenter tip shapes. Even though no puncture was achieved, the sharpest tools (pointed and wedge indenter) did leave a clearly visible imprint. For the *brittle* models, the ringed tip shape was the most effective, whereas the pointed indenter tip shape showed the least effective results. For the non-pointed indenter tip shapes tested, no distinction could be made regarding their efficiency or effectiveness on fracturing models; all performed similarly well in fracturing the brittle material models and were able to puncture reference models, with more ductile characteristics. Furthermore, the “open” shapes (*hollow spherical* and *ringed*) performed comparably to the “closed” shapes (*stamp*, *spherical*, and *wedge*), while being advantageous for future clinical prototypes in which a guidewire may need to be guided through the crossing instrument. Due to the uncertainty of the material characteristics of real CTOs, it is difficult to provide a recommendation about which tip shape is the most suitable for CTO crossing. Nonetheless, it can be suggested that the pointed indenter tip shape may be the least appropriate for fracturing CTOs, as these are usually heavily calcified and thus brittle.

During puncture some minor chips of the CTO proximal cap model were formed, which are potentially harmful, as they can lead to stroke in the smaller blood vessels of the brain, for example. In order to minimize this risk, the device should only be used to penetrate the proximal cap of the CTO; crossing of the CTO body should be executed using a dedicated guidewire. Furthermore, it is recommended that the device is used in combination with a proximal emboli filter or active aspiration.

##### Energy Transfer from the Indenter to the CTO Model

The observed maximum displacements of the CTO models (1.4 mm) were lower than the displacements of over 4 mm reported for guidewires statically pushed against a CTO until puncture is achieved (derived from Thind *et al.* 7). Furthermore, in the control experiment, the average displacement of proximal cap models induced by an almost static (0.1 mm/s) guidewire force was 2.1 mm (*n* = 4). It may, therefore, be argued that the impulse method shows merit to minimize stretch of the blood vessel wall during PCI of CTOs. However, other negative effects due to the impulse should be investigated, such as the risk of blood vessel wall damage as a result of a direct hit or damage due to device failure, in which, for example, the indenter or spring disconnect from the device.

In our experiments, the amount of kinetic energy lost during the indenter strike was large (kinetic energy before and after collision were determined at 2.2 mJ and 0.1 mJ, respectively), indicating that the collisions with the CTO models were highly inelastic, albeit not purely inelastic, since up to 5.4% of the energy dissipated in the environment. To limit the displacement of the CTO and associated energy dissipation, the velocity of the indenter should be increased, allowing the inertia and damping of the CTO and its environment to provide a higher counterforce to the applied impulse than in the current design. This could be achieved by diminishing the effect of dry and viscous friction between the moving parts, by increasing the actuation force, and by minimizing the indenter mass. Furthermore, in a future experiment, the energy dissipation to the environment should be estimated by measuring the energy absorbed by the CTO proximal cap model on a fixed surface.

The higher bounce velocity of the indenter and CTO model displacement observed in the cases of successful CTO model puncture compared to the cases when no puncture was achieved can be explained by two possible phenomena. First, it is likely that in the cases of successful puncture, tilting of the two parts of the proximal cap model after fracturing has led to a more concentrated distribution of the impact force on the gelatin, leading to a larger displacement as compared to the cases of no puncture. Second, in the case of puncture less energy is likely to be lost in the proximal cap model, compared to a non-puncture case.

### Design Recommendations

The most important redesign step of the prototype concerns the transformation of the rigid design into a flexible clinical instrument. In order to allow for navigating towards the lesion site, a flexible shaft will need to be placed between the tip section and the handle. The flexible shaft should be axially incompressible or force neutral (such as a Bowden cable) to prevent axial compression and movement during activation of the tip section. Furthermore, the inner locking core needs to be replaced by a flexible cable that runs from the distal tip of the device, through the flexible shaft, towards the handle. To actuate the gripper, a flexible axially stiff tube or cables should also run through the shaft of the device. Additionally, the rigid tip parts such as the outer shaft, gripper, position block, and indenter need to be redesigned to allow for bending motion. For this purpose, the outer shaft can be replaced by a flexible shaft similar to a catheter, a flexible cable can replace the connection between the indenter tip and stop, and the position block and the gripper can contain joints to allow for bending motions while restricting axial compression or extension. Moreover, compression of the spring during actuation may straighten the previously bend catheter shaft, potentially resulting in undesired pressure on, and thus damage of, the arterial wall. The addition of steering cables can possibly prevent or counteract this effect by retaining the bend shape of the catheter during loading and unloading. Another important issue that needs to be addressed is that of the loss of the indenter caused by failure of the connection with the indenter stop. By adding a second stop at the distal end of the catheter, the risk of indenter loss may be minimized. Finally, to allow for hand-held operation of the prototype, the enveloping box needs to be redesigned. The handle can, in theory, be 3D-printed in any arbitrary shape to meet the needs and preferences of the operator, as long as it contains an insert for the loading, locking, and trigger mechanism.

The prototype has proven effective in puncturing the CTO models. However, it is recommended that the indenter velocity is increased to further decrease the CTO displacement and increase the puncture effectiveness. For this purpose, the effect of the BMF viscosity should be minimized by, for example, adding a sealing that prevents blood from entering the prototype. Furthermore, stiffer spring designs should be investigated, and the effect of friction within the device tip should be minimized by, for example, using low friction coefficient material combinations. Finally, different types of output characteristics need to be investigated, including different vibrating motions, to determine the most optimal output characteristic for achieving puncture of the different CTO models.

To allow for atraumatic navigating through the vasculature and account for diameter differences between patients, the prototype should be further miniaturized. Current clinically available dedicated CTO devices have a diameter of in between Ø0.43 and Ø1.5 mm, so miniaturization to at least the Ø1.5 mm is preferred. Miniaturization of the prototype is relatively easy to achieve due to the small number of parts (14) and low complexity of the prototype. The main challenge lies in the miniaturization of the gripper and its counterpart, the indenter stop.

### Limitations of this Study

The development of an accurate and reproducible coronary CTO model is a complex undertaking and, up to today, has not produced a representative replica of a CTO. This is mainly because simulating calcification and the inflammatory component is difficult. In order to evaluate the device performance, it was chosen to use an artificial CTO model. This CTO model allowed us to test the device under controlled conditions, which is not possible using an animal CTO model. However, image analysis of the CTO models to further characterize them is needed. This will determine how well the artificial CTO models mimic real CTOs. In future research it is recommended that the device is tested in an animal CTO model, such as the one described by Thind *et al*.,^7^ to further determine its effectiveness.

## Conclusion

The low success rates of endovascular revascularization of CTOs can be mainly attributed to the inability to cross the proximal caps of heavily calcified CTOs. In an effort to pursue improved crossing ability, we investigated a new crossing method in which an impulse is applied onto the CTO. Using an impulse to penetrate the proximal cap of heavily calcified CTOs is proven to be advantageous as it increases the buckling resistance of the tool, minimizes movement of the CTO, and can potentially decrease the penetration load. A proof-of-principle prototype was developed that uses a spring-loaded indenter with interchangeable tip shapes and a compliant reload mechanism. The prototype was evaluated in terms of its mechanical performance and puncture effectiveness on CTO models made of gelatin and calcium. From this experiment it became clear that the proposed impulse prototype outperforms currently available dedicated CTO guidewires when it came to puncture performance. A maximum mean indenter velocity of 3.6 m/s, translating to an indenter momentum of 1.33 mNs, and a maximum mean impact peak force of 9.2 N were measured in BMF. This impact peak force is well over what can be delivered by dedicated CTO guidewires, as well as the 1.52 N puncture force previously measured in rabbit femoral CTOs of 15 weeks of age, and should thus be sufficient to penetrate real CTOs.^7^ Furthermore, the displacement of the CTO model was significantly less with the proposed prototype than in a control experiment with a rigid rod and in the animal CTO model of Thind *et al*.^7^ In contrast to current CTO devices and guidewires, the prototype was most effective and efficient on the *brittle* CTO models using the *spherical*, *hollow spherical with guidewire passage*, and *ringed* tip shape. Future developments will be focused on developing a smaller (Ø1 mm), faster, and flexible clinical prototype that allows for penetrating both soft and hard CTOs with minimal tissue deformation and energy dissipation. This prototype will be guided over a guidewire through the vasculature towards the occlusion in one of the coronaries where it can aid in penetrating the proximal caps of the most heavily calcified CTOs.

## Figures and Tables

**Figure 16:**
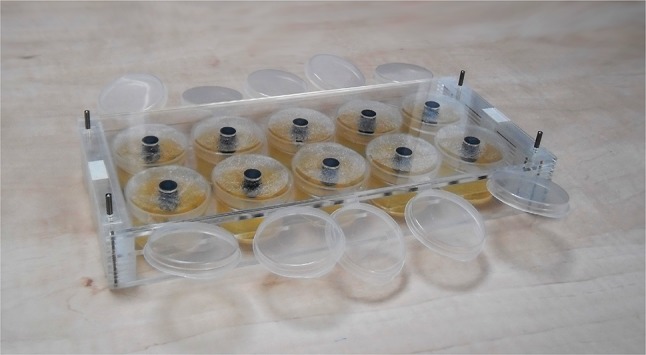
The mold for creating the environment models. The mold consists of laser-cut disks in between which 10 circular containers can be placed. The lid of the mold, in which several rods are placed, creates the notches.

**Figure 17:**
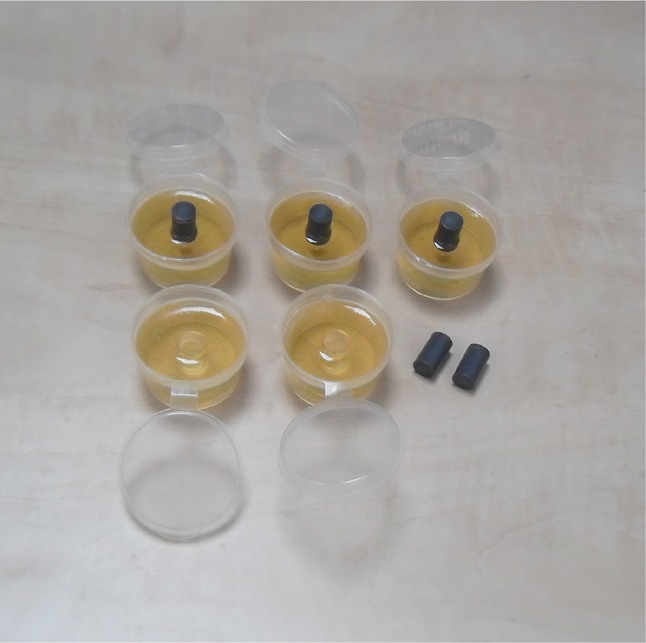
Environment models with notches in the center.

**Figure 18:**
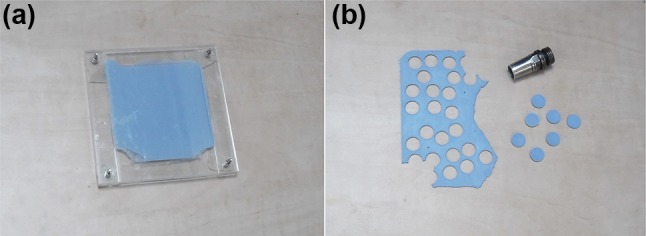
The proximal cap model. Left: Mold for the proximal cap models, to create the desired 1 mm material thickness, filled with the gelatin/plaster cast mixture Right: the complete molded slice of material (left), thin-walled tube to press the circles (top right), and final proximal cap models (lower right).

## Appendix: CTO Model Preparation

In this appendix, the procedure that was followed to prepare the environment models (representing the surrounding cardiac tissue) and proximal cap models is described.

### Environment Models

The following steps were followed to prepare the environment models:

1. Weigh a specific amount (depending on the quantity you need) of gelatin (*Sheet gelatin*, Dr. Oetker, Bielefeld, Germany).
2. Put the gelatin sheets in a bath of cold fresh water (∼15 °C), and leave to soak for 5 min.
3. Take the gelatin sheets from the bath and wring them meticulously before putting them in a plastic container.
4. Weigh the new (soaked) mass of the gelatin sheets, and add an amount of cold clear water to create the 25 wt% of gelatin.
5. Put the gelatin-filled container in a bath of warm water (∼50 °C) and stir gently until the content is completely liquid.
6. Take the container out of the bath and leave it to rest for approximately 15 min, during which all bubbles will float to the surface and can be removed from the mixture.
7. Pour the gelatin mixture in a small container that is placed in the mold illustrated in Fig. 16.
8. Close the mold with the lid to create a notch within the gelatin volume.
9. Leave the gelatin-filled containers for approximately 1 h in a refrigerator (∼2 °C), before gently removing the lid of the mold from the gelatin (see Fig. 17).
10. Close the lid of container and leave the gelatin mixture to harden in the refrigerator for another 24 h.

Note: To ensure constant tissue stiffness, the tissue model is to be used directly from the refrigerator (<1 h).

### Proximal Cap Models

The following steps were followed to create the proximal cap models:

1. Prepare the gelatin mixture as described in step 1–6 from the previous procedure (surrounding tissue model).
2. Weigh a specific amount (depending on the quantity you need) of calcium sulfate (*SHERAALPIN Hartgips hellblau*, SHERA Werkstoff-Technologie GmbH & Co. KG).
3. Put the powder in a small plastic cup and add a correct amount of gelatin mixture into the cup.
  - For the *ductile* proximal cap model: 50 wt% calcium sulfate powder and 50 wt% gelatin mixture;
  - For the *reference* proximal cap model: 75 wt% calcium sulfate powder and 25 wt% gelatin mixture;
  - For the *brittle* proximal cap model: 77 wt% calcium sulfate powder and 23 wt% clear water.
4. Stir the mixture gently, while holding the cup in a bath of warm water (∼50 °C), until a homogeneous mixture has formed.
5. Pour the mixture onto the cap-model mold, and close with force to get a 1 mm thick slice of proximal cap models (see Fig. 18).
6. Leave the filled cap-model mold for 20 min in a refrigerator (∼2 °C) to stiffen.
7. Remove the cap-model mold from the refrigerator and open it to take out the formed slice of (at this moment still flexible) material.
8. Press the small circles from the material with the help of a thin-walled tube, as shown in Fig. 18.
9. Pack the models into cling film, and leave them for another 24 h in the refrigerator.

Note: To ensure constant material stiffness, the model is to be used directly from the refrigerator (< 1 h).
